# Pepper mild mottle virus as a potential indicator of occupational exposure to airborne viruses in wastewater treatment plants

**DOI:** 10.1093/annweh/wxaf020

**Published:** 2025-05-24

**Authors:** Anna Jacobsen Lauvås, Pål Graff, Anani K Afanou, Caroline Duchaine, Marc Veillette, Mette Myrmel, Anne Straumfors

**Affiliations:** Department of Occupational Toxicology, National Institute of Occupational Health, Gydas vei 8, Majorstuen 0363, Oslo, Norway; Virology unit, Department of Paraclinical Sciences, Faculty of Veterinary Medicine, Norwegian University of Life Sciences, Elizabeth Stephansens v. 15, 1433 Ås, Norway; Department of Chemical Work Environment, National Institute of Occupational Health, Gydas vei 8, Majorstuen 0363, Oslo, Norway; Department of Occupational Toxicology, National Institute of Occupational Health, Gydas vei 8, Majorstuen 0363, Oslo, Norway; Département de Biochimie, de Microbiologie et de Bio-informatique, Faculté́ des Sciences et de Génie, Université Laval, Pavillon Alexandre-Vachon, 1045 Av. de la Médecine, Québec, QC G1V 0A6, Canada; Centre de Recherche, Institut Universitaire de Cardiologie et de Pneumologie-Université Laval, 2725 Ch Ste-Foy, Québec, QC G1V 4G5, Canada; Virology unit, Department of Paraclinical Sciences, Faculty of Veterinary Medicine, Norwegian University of Life Sciences, Elizabeth Stephansens v. 15, 1433 Ås, Norway; Department of Occupational Toxicology, National Institute of Occupational Health, Gydas vei 8, Majorstuen 0363, Oslo, Norway

**Keywords:** AdV, air sampling, bioaerosol, ddPCR, InfA, NoV, PMMoV, WWTP

## Abstract

Wastewater is a known carrier for human pathogenic viruses, with seasonal variations in concentrations, and wastewater treatment plant (WWTP) workers are a potentially overlooked occupational group regarding exposure to secondary aerosolized viruses. Exposure assessment of airborne pathogens is complicated by a lack of universal markers of viruses, no standardized sampling protocol, and challenges in detecting extremely low-abundant targets. In this study, we evaluate the risk of workers’ exposure to 4 pathogens, Adenovirus, Norovirus GI and GII, and Influenza A and the Pepper mild mottle virus (PMMoV) as an indicator for aerosolized viruses from wastewater, in 3 WWTPs in the Oslo region, Norway. We collected personal and stationary air samples in summer and winter and used digital droplet PCR (ddPCR) to enable the detection of low-abundant targets. Pathogenic viruses were detected in 22% of all samples, with similar detection rates in personal and stationary samples, with a maximum concentration of 762 genome copies/m^3^ air. PMMoV was detected in 69% of all samples, with concentrations ranging from 28 to 9703 genome copies/m^3^ air. The pathogens and PMMoV were most frequently detected at the grids, biological cleansing, sedimentation basins, and sludge treatment/de-watering stations, and were associated with tasks such as flushing, cleaning, and maintenance of the same workstations. Overall, the concentration of pathogens and PMMoV in the air was low, but there is a potential for high point exposure which may pose a risk to workers’ health and is increased by the nature of the workers’ tasks. PMMoV may be a promising tool for assessing the overall potential for viruses with human waste origin aerosolized from sewage. To strengthen this indicator-based approach to occupational exposure assessment, we recommend validating PMMoV along with other potential indicators. Validation should include evaluating the correlation between these indicators and pathogens in both wastewater and bioaerosols.

**What’s Important About This Paper?**

This study demonstrates the presence of aerosolized viruses in wastewater treatment plants, confirming that workers may be exposed during their tasks, despite implemented risk mitigation strategies such as improved ventilation and encapsulation of high-risk workstations. Owing to the myriad viruses that may be present, pepper mild mottle virus is proposed as a potential tool to assess the exposure of viruses aerosolized from sewage.

## Introduction

Occupational groups at risk for contracting viral infections through their work have primarily been considered to include health personnel and others exposed to primary aerosolized viruses from infected individuals. Wastewater, however, is a well-known carrier of various human pathogenic viruses such as Norovirus (NoV), human Adenovirus (AdV), Rotavirus, Hepatitis A and E virus, poliovirus, and lately, SARS-Cov-2 (Lewis et al. 2010; Grondahl-Rosado et al. 2014; De Schryver et al. 2015; Duintjer Tebbens et al. 2017; Eftim et al. 2017; Kuodi et al. 2020). During wastewater treatment processes, bioaerosols are generated, which may contain microorganisms such as bacteria and fungi, as well as viruses (Verreault et al. 2008). As a result, workers in wastewater treatment plants (WWTPs) may be at risk of exposure to a wide range of viral pathogens, particularly during tasks involving direct contact with sludge or wastewater (Uhrbrand et al. 2011; De Schryver et al. 2015). Previous reports indicate that WWTP workers have an increased risk of developing symptoms such as diarrhea and nausea, headache, cough with phlegm, unusual tiredness, and joint pains (Lundholm and Rylander 1983; Rylander 1999). Some of these symptoms are similar to those caused by infections with AdV serotypes A, D, F, and G (gastroenteritis), AdV B, C, and E (upper and lower respiratory tract infections, and conjunctivitis) (Heim 2021), NoV (gastroenteritis) (Chan et al. 2017), Respiratory Syncytial Virus (RSV) (upper respiratory tract infection) (Collins et al. 2013), or Influenza A, B or C (respiratory tract infection) (Moghadami 2017). Several studies have detected viruses that are transmitted via the fecal-oral route in WWTP air, such as NoV GI and NoV GII (Uhrbrand et al. 2011; Courault et al. 2017; Pasalari et al. 2019; Stobnicka-Kupiec et al. 2022). AdVs have also been detected at concentrations ranging from 933 (Carducci et al. 2016) to 4700 viral genome copies per m^3^ air (gc/m^3^) (Brisebois et al. 2018).

Common viral infections exhibit seasonal variation in prevalence, which is reflected in fluctuating virus concentrations in sewage (Grondahl-Rosado et al. 2014; Amato et al. 2023). Virus concentrations in WWTP air will also depend on the degree of aerosolization from the wastewater, and the sampling strategy further influences these viruses’ detection and quantification. Filter-based air sampling is advantageous in continuous, low airflow sampling, but is associated with the desiccation of samples and inconsistent recovery (Burton et al. (2007); (Uhrbrand et al. 2018). High-volume air sampling enables the collection of large air volumes, but the force of impactors may compromise the detection of viral aerosols (Stobnicka-Kupiec et al. 2022), and the use of water-based cyclonic samplers may result in sample loss due to re-aerosolization (Lemieux et al. 2019). Environmental detection of viruses is further complicated by the lack of universal biomarkers, unlike bacteria and fungi, which can be detected and quantified using the 16S rRNA and ITS (Internal Transcribed Spacer) genes conserved sequences. Various bacteriophages have been used as proxies for viral pathogens assessment in WWTP and swine-building air (Heinonen-Tanski et al. 2009; Létourneau et al. 2025). However, bacteriophage levels fluctuate throughout the wastewater treatment process and can increase in the presence of host bacteria (García-Fontana et al. 2020). They may therefore not accurately reflect the aerosolization of viruses with human waste origin, which are degraded through the treatment process (Hamza et al. 2011, 2019; Tandukar et al. 2020). An alternative proxy could be the Pepper mild mottle virus (PMMoV), a rod-shaped, single-stranded RNA virus, belonging to the *Tobamovirus* family. This plant virus infects hot, bell, and ornamental peppers and is found more consistently and in higher concentrations in human feces compared to other animals (Kitajima et al. 2018). PMMoV is geographically widespread and has been detected in Australia, North- and South America, the Middle East, Europe, and Norway, with concentrations ranging from 4 to 12 log_10_ gc/L in sewage. PMMoV has also been reported to coincide with NoV, AdV, SARS-CoV-2, Sapovirus (SV), and HPV (Kitajima et al. 2014; Hamza et al. 2019; Ahmed et al. 2021, 2022, 2023; Rashed et al. 2022; Burnet et al. 2023). Due to its widespread presence in sewage, and its association with human feces, PMMoV is now commonly used as a normalizing factor for population prevalence of SARS-CoV-2 in wastewater surveillance studies (Hyllestad et al. 2022; Amato et al. 2023).

This study is part of a larger project where we aim to characterize airborne exposure to viral- and bacterial-laden bioaerosols in selected Norwegian WWTPs. The present study aims to evaluate the risk of workers’ exposure to selected human pathogenic viruses and to evaluate the use of PMMoV as an indicator of viral aerosolization from sewage in selected Norwegian WWTPs. We also aim to assess differences between seasons and plants and to identify workstations and tasks associated with increased risk of exposure.

## Materials and methods

### Study design

Personal and stationary air sampling was performed in three WWTPs (A, B, and C) in the Oslo fjord region in the period December 2022—June 2023. Plants A and B are large, industrial processing plants, built into mountain halls, providing advanced wastewater treatment, servicing 500 000 (A) and 800 000 (B) inhabitants in Oslo city and the surrounding municipalities. Plants A and B were visited twice in winter, and twice (A) and once (B) in summer. Plant C is located further south along the Oslo fjord, serving less than 10 000 inhabitants, and was included due to its small size. This plant provides basic wastewater treatment, is staffed by only one permanent employee, and was visited once during winter. The pools containing stagnant wastewater were located in separate rooms in plants B and C, whereas in plant A, they were in larger, open halls equipped with point ventilation near the wastewater surface.

Personal exposure measurements were obtained from workers with voluntary participation at each sampling day. The workers were equipped with personal air samplers within their inhalation zone, max. 30 cm from their mouth and sampling was conducted through a full shift of approx. 4 to 6 hours. On each sampling day, the workers were asked to provide information about the duration and type of tasks performed at each workstation, working hours, and time spent within the plant.

The list of workstations, wastewater treatment processes, and description of associated tasks covered during the air sampling are summarized in Table 1 along with the number of collected personal and stationary samples per plant. The workstations for stationary sampling were selected based on the regularly performed tasks connected to them or whether they involved direct or indirect contact with wastewater or sludge. The air treatment room in plant A filtered all air collected from the ventilation system and this unfiltered air was considered a potential worst-case setting for exposure within plant A.

**Table 1.** Description of workstations and the number of stationary air samples collected per workstation and plant (A, B, and C). The table outlines the wastewater treatment processes, and the associated tasks regularly performed by employees at each workstation. NA: not applicable; the workstation was not part of the plant’s treatment process.

Personal samples	Samples (*N*)				
Plant	A	B	C		
Summer	2	4	0		
Winter	8	16	1		
Sum	10	20	1		
Sum personal	31				
**Stationary samples**	**Samples (*N*)**				
**Plant\** **Workstation**	**A**	**B**	**C**	**Wastewater treatment process**	**Regularly performed tasks**
Water intake	3			Intake from the tunnel system	None (A), flushing of floors (C)
Grids	5	6	2	Removal of solid debris from wastewater	Flushing and manual removal of solid debris
Sedimentation basin	4	4	NA	Separation of sludge and wastewater	None (A), flushing of gutters and floor (B)
Flocculation basin	1		2	Chemical separation of sludge and wastewater	Maintenance and flushing
Sand filter	3		NA	Removal of fine debris	Maintenance, cleaning (A, B)
Biological cleansing	7		NA	Nitrogen removal	None (A), maintenance
Processing hall		3	NA	General processes	Maintenance, repair (B)
Sludge treatment/ de-watering	2	5	4	Separation of water from sludge. Storage of sludge until transport (containers)	Flushing of grit chambers, presses (B) or centrifuges
Air treatment	2		1	Filtration of air from ventilation system before release to environment	Flushing and maintenance
Not noted	1	1			
Sum	28	19	9		
Sum stationary	56				

### Ethical considerations and selection of participants

The study was approved by the Regional Committee for Medical and Health Research Ethics of South-East Norway (REK Case no. 314217). Participating workers were chosen in cooperation with the health and safety managers at each plant, and based on the nature of their work, which involved proximity to the plant processes associated with potential exposure. Participation was voluntary and informed consent was obtained before the field sampling.

### Air sampling

Inhalable bioaerosol fractions (mean 1.3 m^3^, Supplementary Table S3) were collected with polycarbonate track-etched filters (PCTE, Pore size 1 µm, 37 mm, GVS North America) mounted in conical inhalable sampler (CIS) cassettes (JS Holdings, Hertfordshire, UK) and operated at an airflow of 3.5 L/min, driven by either an Apex2 pump (Casella UK, Wolseley Rd, Kempston, Bedford, UK) or a GSA 5200 pump (GSA, Messgerätebau GmbH, Ratingen, Germany). CIS cassettes were used for both personal and stationary sampling and all pumps were calibrated before use.

High-volume air samples (2 to 8 m^3^, Supplementary Table S3) were collected with the Coriolis µ Microbial Sampler (Bertin Technologies, France) at selected workstations during active, manual labor. The sampler was run at 200 L/min and collected air into 12 to 15 mL of sterile, nuclease-free PBS (1X, pH 7.4, Invitrogen). Coriolis samples were collected in single and multiple 10-min intervals by accumulative sampling, followed by pooling to increase the sampled air volume at each workstation, based on the approach by Rufino de Sousa et al. (2020). All stationary samplers were positioned approx. 1.5 m above the floor, except at the station near the processing hall in plant B, where they were placed on an industrial mesh floor covering running wastewater, approx. 30 to 50 cm above the water surface. This location was selected as a potential worst-case scenario due to its proximity to the wastewater.

In total, 87 samples were collected, including 61 filter samples (31 personal and 30 stationary), and 26 Coriolis samples. Upon completion of sampling, the Coriolis cones were closed, and all filter cassettes were stored in individual, clean zip lock bags. All samples were stored on ice during transportation to the laboratory for immediate processing. The arithmetic average and range of collected air volumes and sampling time are presented in Supplementary Table S3.

### Measurements of relative humidity and temperature

Relative humidity (%RH) and temperature were monitored for selected workstations and workers, using environmental sensors developed in-house. The sensors recorded relative humidity, temperature, atmospheric pressure, and an uncalibrated measurement of volatile compounds using a BME680 sensor (Adafruit, USA), coupled with the time and date using a FeatherWing—RTC + SD module (Adafruit, USA). Outdoor temperature data were retrieved from the Norwegian Meteorological Institute at www.yr.no by identification of the closest meteorological measuring station to each plant on each sampling day. A descriptive overview of %RH, temperature, and recovery of liquid samples is given in Supplementary Table S4.

### Sample treatment and nucleic acid extraction

Total nucleic acids (tNA) were lysed from the filter samples (*n* = 57) by adding 1 mL PM1 lysis buffer with 2% 2M dithiothreitol (DTT) directly onto the filter. The volumes of Coriolis (*n* = 26) samples were measured before it was concentrated using tangential flow filtration with a 30 kDa cutoff (Amicon Ultra Centrifugal Filter, Merck/Millipore, Darmstadt, Germany) at 4500 G for 15 min at 4°C, adapted from the protocol by Brisebois et al. (2018). In the first winter sampling in plant B, 3 Coriolis samples were divided into 400 µL aliquots. Additionally, 4 filter samples were eluted with a protocol modified from Alonso et al. (2017). The filters were transferred to a nuclease-free falcon tube with 3 mL PBS (1X, Gibco), inverted 10 times, allowed to set for 5 min, and inverted an additional 10 times, before sonication in an ultrasonic bath with sweep function for 5 min (Fisherbrand, FB15052, 50/60 Hz) and divided into 400 µL aliquots. Aliquots and concentrated samples were lysed in PM1 lysis buffer with 2% DTT and stored at -20 °C. Nucleic acids were extracted into 50 µL of water using the AllPrep Power Viral DNA/RNA Kit (Qiagen, Hilden, Germany), following the manufacturer’s instructions.

### Quantification of viral pathogens

Selection of viruses were based on three criteria: (i) the virus had previously been detected in wastewater or WWTP aerosols, or (ii) the virus could cause symptoms observed in WWTP workers, and (iii) the virus was not part of a public or occupational health vaccination program (Hepatitis A and B, SARS-Cov-2, and rotavirus were excluded). Four viruses were selected: Adenovirus, Influenza A, and Noroviruses in genogroups I and II.

RNA was reverse transcribed using the qScript cDNA Synthesis kit (Quantabio, Massachusetts, US) according to the manufacturer’s instructions. The concentration of each virus genome was quantified using digital droplet PCR (ddPCR) with 5 µL template. The qPCR assays (Supplementary Table S1) were optimized for ddPCR by a temperature gradient and concentration matrix (data not shown). Each assay was run with 900 nM forward and reverse primers, 250 nM probe, and 1X Supermix for Probes (no dUTP) (BioRad, US), following the manufacturer’s protocol with an initial denaturation at 95 °C for 10 min, followed by 45 cycles of 94 °C for 30 s and 55 °C for 1 min, and a final denaturation at 98 °C for 10 min. Each sample was run in 2 to 4 technical replicates, with a minimum of 2 positive and 2 negative (no template, NTC) controls.

The threshold was individually set for each target by repeatedly evaluating the positive and negative controls, ensuring optimal separation of positive and negative droplets. Due to the specificity of the primers, the assay limit of detection (aLOD) was set to 1 positive droplet, equivalent to 292 genome copies per liquid sample or filter. The detection limit in different air volumes and representative amplitude plots are provided in Supplementary Table S2 and Figure S1. Wells with <10 000 accepted droplets were excluded from further analysis, according to the manufacturer’s instructions (Biorad, US), resulting in the exclusion of one stationary sample for each of the targets AdV, NoV GII, InfA, and PMMoV (4% of the total dataset). All wells with ≥10 000 accepted droplets were considered quantifiable; thus, all detectable samples were also quantifiable, and the genome concentration for each sample was calculated as the average concentration across all technical replicates. Plate runs with positive droplets in the NTC were excluded and could not be re-run due to limitations in the sample material, resulting in the exclusion of 11 PMMoV samples (11%). After applying all quality criteria, results were obtained from the following number of samples: AdV (*n* = 82), NoV GI (*n* = 63), NoV GII (*n* = 68), InfA (*n* = 68), and PMMoV (*n* = 74).

### Data analysis

Data management and visualization were executed in R/RStudio (R version 4.3.2) using dplyr and tidyr for data management (Wickham et al. 2023, 2024), and ggplot2 and cowplot for data visualization (Wickham et al. 2016; Claus 2024). Data analysis was performed with STATA (StataCorp. 2023. *Stata Statistical Software: Release 18*. College Station, TX: StataCorp LLC). All results are presented as the arithmetic mean of at least 2 wells, except for 10 PMMoV samples (11%) and one NoV GII sample (1.5%), which are based on one technical replicate. Plant, season, and sampler differences were evaluated with the Kruskal–Wallis nonparametric test on the overall concentration of all samples and for positive samples only. All group comparisons were performed with stratification by sampler. A logistic regression model was fit to evaluate detection (0/1) with the predictors plant, season, sampler, and sample type. Due to lognormally distributed data and lack of suitable imputation strategies for negative values below LOD, quantile regression was employed to detect differences in concentrations between PMMoV and the summed concentration of pathogens. Each regression model and its output were evaluated based on the overall *R*^2^, and the 95% confidence intervals, standard errors, and *P*-values for each level of predictor. *P*-values smaller than 0.05 were considered statistically significant.

## Results

### Viruses in WWTP air

We detected InfA, NoV GII, NoV GI, or AdV in 18 out of 82 samples (22%). There was no statistically significant difference in detection rate between personal and stationary samples, with 26% and 21% samples positive for at least one pathogen, respectively (Table 2 and Table 3). In three personal samples, 2 pathogens were detected independently. In 2 of these samples, both collected in plant A, NoV GII, and InfA were detected at a maximum summed concentration of 621 gc/m^3^. In the third, from plant B, AdV co-occurred with NoV GI at a summed concentration of 264 gc/m^3^. The highest summed concentration of 621 gc/m^3^ was lower than the highest concentration of an individual pathogen in stationary samples (756 gc/m^3^ NoV GII, Table 3). However, we could not detect any significant difference in concentrations between personal and stationary samples for either pathogen. PMMoV was detected in 51 out of 74 (69%) of all samples, in concentrations ranging from 28 to 9703 gc/m^3^, with the highest concentrations observed in plant B in winter. Overall, there was no statistically significant difference in virus air concentration or detection frequency between plants, but PMMoV was found in statistically significantly higher concentrations than the summed pathogens both overall, and within personal and stationary samples (*P* <0.001, coefficient ± SE: 126 ± 24.6 [78.1 to 173.9]).

**Table 2.** Personal measurements of airborne viruses; Pepper mild mottle virus (PMMoV), Adenovirus (AdV), Influenza A (InfA), Norovirus (NoV) GI and GII. Viruses were quantified by ddPCR, and results are presented for all samples and stratified by plant and season. The data are presented as detection rates (positive samples per total samples (N) and %), statistics for concentrations in all samples (arithmetic mean, median, and interquartile range), and for positive samples only (arithmetic mean and observed range). Virus concentrations are expressed as genome copies (gc)/m^3^ air. ND = not detected.

			All samples (gc/m^3^)		Positive samples (gc/m^3^)	
	Virus	Pos/N (%)	Mean ± SD	Median [25^th^ –75^th^]	Mean ± SD	Min–max
**All personal**	AdV	4/27 (15)	12 ± 32	0 [0–0]	82 ± 40	38–120
	InfA	2/26 (8)	23 ± 91	0 [0–0]	294 ± 224	135–452
	NoV GI	1/27 (4)	5 ± 28	0 [0–0]	144	-
	NoV GII	3/27 (11)	19 ± 58	0 [0–0]	175 ± 48	130–226
	Sum pathogens	7/27 (26)	59 ± 142	0 [0–19]	226 ± 209	38–621
	PMMoV	20/30 (67)	160 ± 136	126 [0–276]	240 ± 89	110–368
Plant A	AdV	0/6 (0)	ND	ND	ND	-
	InfA	2/6 (33)	98 ± 182	0 [0–101]	294 ± 224	135–452
	NoV GI	0/6 (0)	ND	ND	ND	-
	NoV GII	2/6 (33)	66 ± 104	0 [0–127]	198 ± 40	169–226
	Sum pathogens	2/6 (33)	164 ± 267	0 [0–271]	491 ± 184	361–621
	PMMoV	5/10 (50)	108 ± 132	58 [0–200]	216 ± 100	116–342
Plant B	AdV	4/20 (20)	16 ± 37	0 [0–0]	82 ± 40	38–120
	InfA	0/20 (0)	ND	ND	ND	-
	NoV GI	1/20 (5)	7 ± 32	0 [0–0]	144	-
	NoV GII	1/20 (5)	6 ± 29	0 [0–0]	130	-
	Sum pathogens	5/20 (25)	30 ± 67	0 [0–10]	120 ± 89	38–264
	PMMoV	15/20 (75)	186 ± 133	246 [82–-304]	248 ± 87	110–368
Plant C	AdV	0/1 (0)	ND	ND	ND	-
	NoV GI	0/1 (0)	ND	ND	ND	-
	NoV GII	0/1 (0)	ND	ND	ND	-
	Sum pathogens	0/1 (0)	ND	ND	ND	-
Summer	AdV	2/6 (33)	25 ± 44	0 [0–28]	74 ± 51	38–110
	InfA	0/6 (0)	ND	ND	ND	-
	NoV GI	0/6 (0)	ND	ND	ND	-
	NoV GII	1/6 (17)	22 ± 53	0 [0–0]	130	130–130
	Sum pathogens	3/6 (50)	46 ± 59	19 [0–92]	93 ± 48	38–130
	PMMoV	4/6 (67)	161 ± 143	186 [31–-252]	242 ± 90	124–342
Winter	AdV	2/21 (10)	8 ± 29	0 [0–0]	89 ± 44	58–120
	InfA	2/20 (10)	29 ± 104	0 [0–0]	294 ± 224	135–452
	NoV GI	1/21 (5)	7 ± 31	0 [0–0]	144	–
	NoV GII	2/21 (10)	19 ± 60	0 [0–0]	198 ± 40	169–226
	Sum pathogens	4/21 (19)	62 ± 159	0 [0–0]	326 ± 234	58–621
	PMMoV	16/24 (67)	160 ± 137	126 [0–284]	240 ± 92	110–368

**Table 3.** Stationary measurements of airborne viruses; Pepper mild mottle virus (PMMoV), Adenovirus (AdV), Influenza A (InfA), Norovirus (NoV) GI and GII. The viruses were quantified with ddPCR, and the results are presented stratified by air sampler. The data are presented as detection rates (positive samples per total samples (N) and %), statistics for concentrations non-normalized to air volume (arithmetic mean), and normalized to the sampled air volume (arithmetic mean and median and interquartile range), for all samples and for positive samples only. The observed range is provided for positive samples. Virus concentrations are expressed as genome copies per sample (non-normalized to air volume) and genome copies per m3 air (normalized to air volume).

			All samples			Positive samples			
			gc/sample	gc/m^3^		gc/sample	gc/m^3^		
	Target	Pos/N (%)	Mean ± SD	Mean ± SD	Median [25^th^ - 75^th^]	Mean ± SD	Mean ± SD	Median [25^th^–75^th^]	Min–max
Stationary CIS cassettes	AdV	1/29 (3)	6 ± 30	4 ± 23	0 [0–0]	160	122	-	-
	InfA	1/26 (4)	7 ± 33	6 ± 31	0 [0–0]	170	158	-	-
	NoV GI	0/27 (0)	ND	ND	ND	ND	ND	ND	-
	NoV GII	3/27 (11)	52 ± 211	32 ± 146	0 [0–0]	464 ± 539	289 ± 406	94 [56–425]	18–756
	Sum pathogens	5/29 (17)	59 ± 205	40 ± 14	0 [0–0]	345 ± 415	230 ± 299	122 [94–158]	18–756
	PMMoV	19/28 (68)	601 ± 1939	661 ± 1960	130 [0–252]	885 ± 2318	974 ± 2333	180 [130–360]	97–9703
Coriolis	AdV	5/20 (25)	59 ± 132	36 ± 105	0 [0–4]	237 ± 173	143 ± 184	74 [25–136]	18–460
	InfA	0/5 (0)	ND	ND	ND	ND	ND	ND	-
	NoV GI	0/4 (0)	ND	ND	ND	ND	ND	ND	-
	NoV GII	1/8 (12)	20 ± 57	3 ± 10	0 [0–0]	161	27	-	-
	Sum pathogens	6/20 (30)	67 ± 133	37 ± 105	0 [0–20]	224 ± 157	123 ± 171	50 [26–120]	18–460
	PMMoV	14/16 (88)	1301 ± 1673	434 ± 760	159 [50–432]	1487 ± 1712	496 ± 796	269 [63–519]	28–3090

ND = Not detected.

Viral pathogens were detected in both personal and stationary samples, and all 4 were detected in winter, whereas only AdV and NoV GII were detected in summer (Supplementary Table S5). The WWTP environment was consistent over time, with relatively constant indoor temperatures across plants and seasons, unaffected by outdoor temperatures. The relative humidity ranged from 20% to 85 % across all plants and seasons (Supplementary Table S4). The overall concentration in the air was low, whereas individual measurements ranged from 18 to 756 gc/m^3^ (NoV GII). Exposure measurements for all samples and positive samples are, therefore, presented separately (Tables 2 to 4). The average summed concentration of pathogens in positive samples was equal in both personal and stationary CIS cassettes and significantly lower than PMMoV in all samples (Tables 2 and 3). The concentration of pathogenic viruses in air was generally lower in the Coriolis than the stationary CIS cassettes, as seen by the median and interquartile range (Table 3). However, there was no significant difference in the detection rate or the concentrations non-normalized to the air volume (genome copy per sample, Table 3).

The recovery of samples from the Coriolis was high, ranging from 63% to 121 % (Supplementary Table S4). There was no significant difference between seasons, regarding the detection rate of viruses overall. However, PMMoV was detected significantly more often in summer than in winter in stationary CIS cassettes. Coriolis trended towards a higher detection rate of PMMoV in winter than stationary CIS cassettes (OR 9.33 [0.89 to 97.62], *P* = 0.062) and there was a weak tendency towards lower detection rates of PMMoV in winter than summer across all sample types and samplers (OR 0.34 [0.11 to 1.03], *P* = 0.057). Seasonal variation in personal samples could not be evaluated due to an insufficient number of positive samples. The concentration estimates of each virus in air are given for each sampler (Table 3), and the detection rates are given for each sampler, season, and plant (Supplementary Table S5). As few pathogens were detected, exposure estimates in stationary samples stratified by plant and season are therefore given for PMMoV and the summed pathogens only in Table 4. Air concentrations in PMMoV across all samples were generally in higher agreement with the concentrations in positive samples only than the pathogens (Tables 3 and 4).

**Table 4.** Stationary measurements of airborne viruses; Pepper Mild Mottle Virus (PMMoV), and the sum of pathogenic viruses. The viruses were quantified with ddPCR, and the results are presented stratified by air sampler. The data are presented as detection rates (positive samples per total samples (N) and %), statistics for concentrations in all samples and for positive samples only (arithmetic mean, median, and interquartile range). The observed range is provided for positive samples. The sum pathogens represent the summarized detection rate and concentrations of Adenovirus, Influenza A and Norovirus GI and GII. Virus concentrations are expressed as genome copies per m^3^ air.

		Target		Summer	Winter	Plant A	Plant B	Plant C
Stationary CIS cassettes	All samples	PMMoV	Pos/N (%)	13/15 (87) *	6/13 (46) *	11/14 (79)	5/9 (56)	3/5 (60)
			Mean ± SD	238 ± 240	1149 ± 2845	228 ± 255	588 ± 1461	2004 ± 4305
			Median [25^th^–75^th^]	138 [106–301]	0 [0–180]	132 [99–326]	124 [0–246]	135 [0–180]
		Sum pathogens	Pos/N (%)	2/15 (13)	3/14 (21)	2/14 (14)	2/10 (20)	1/5 (20)
			Mean ± SD	14 ± 38	67 ± 203	8 ± 25	88 ± 238	32 ± 71
			Median [25^th^–75^th^]	0 [0–0]	0 [0–0]	0 [0–0]	0 [0–0]	0 [0–0]
	Positive samples	PMMoV	Mean ± SD	274 ± 237	2490 ± 3927	291 ± 254	1058 ± 1910	3339 ± 5511
			Median [25^th^–75^th^]	140 [124–356]	226 [177–3423]	140 [117–360]	246 [176–272]	180 [158–4942]
			Range (Min–-max)	97–832	135–9703	97–832	124–4473	135–9703
		Sum pathogens	Mean ± SD	108 ± 20	311 ± 392	56 ± 54	439 ± 448	158
			Median [25^th^–75^th^]	108 [101–115]	158 [88–457]	56 [37–75]	439 [280–598]	-
			Range (Min-–max)	94–122	18–756	18–94	122–756	-
Coriolis	All samples	PMMoV	Pos/N (%)	6/7 (86)	8/9 (89)	6/7 (86)	6/7 (86)	2/2 (100)
			Mean ± SD	237 ± 212	587 ± 994	206 ± 211	771 ± 1076	54 ± 2
			Median [25^th^–75^th^]	228 [56–378]	90 [53–803]	90 [56–339]	388 [135–823]	54 [54–55]
		Sum pathogens	Pos/N (%)	0/2 (ND)	6/18 (33)	2/9 (22)	2/7 (29)	2/4 (50)
			Mean ± SD	ND	41 ± 110	18 ± 45	68 ± 173	25 ± 35
			Median [25^th^–75^th^]	ND	0 [0–23]	0 [0–0]	0 [0–9]	14 [0–39]
	Positive samples	PMMoV	Mean ± SD	276 ± 202	661 ± 1037	240 ± 209	899 ± 1119	54 ± 2
			Median [25^th^–75^th^]	298 [120–383]	200 [55–813]	200 [86–354]	596 [268–833]	54 [54–55]
			Range (Min–max)	28–563	42–3090	28–563	42–3090	53–56
		Sum pathogens	Mean ± SD	ND	50 ± 47	80 ± 78	239 ± 313	50 ± 33
			Median [25^th^–75^th^]	ND	26 [20–62]	80 [53–108]	239 [128–350]	50 [39–62]
			Range (Min-–max)	ND	18–136	25–136	18–460	27–74

ND = not detected. * Significant difference between seasons (*P* < 0.05).

**Figure 1.**
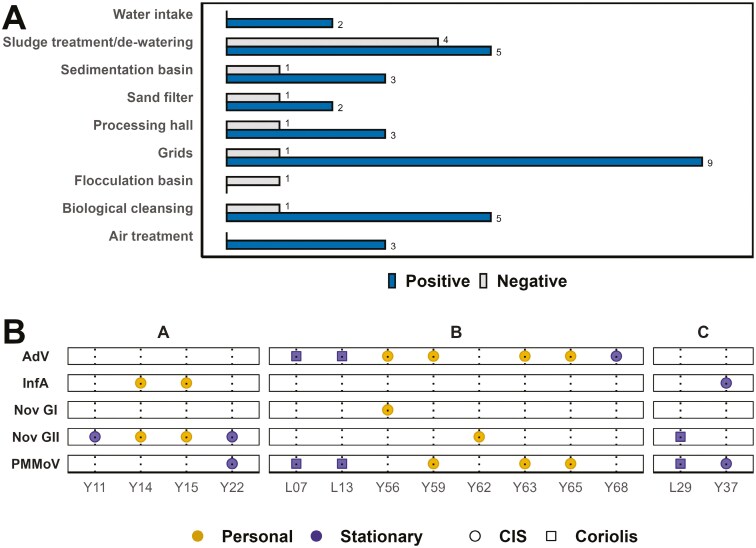
Detection of Pepper Mild Mottle Virus (PMMoV) and viral pathogens in air sampled at wastewater treatment plants. (A) The number of samples positive or negative for PMMoV in stationary samples at various workstations. (B) Individual samples (marked with dots in a vertical line) positive for pathogens, and either positive or negative for PMMoV and Adenovirus (AdV), Norovirus GI, Norovirus GII or Influenza A (InfA).

**Figure 2.**
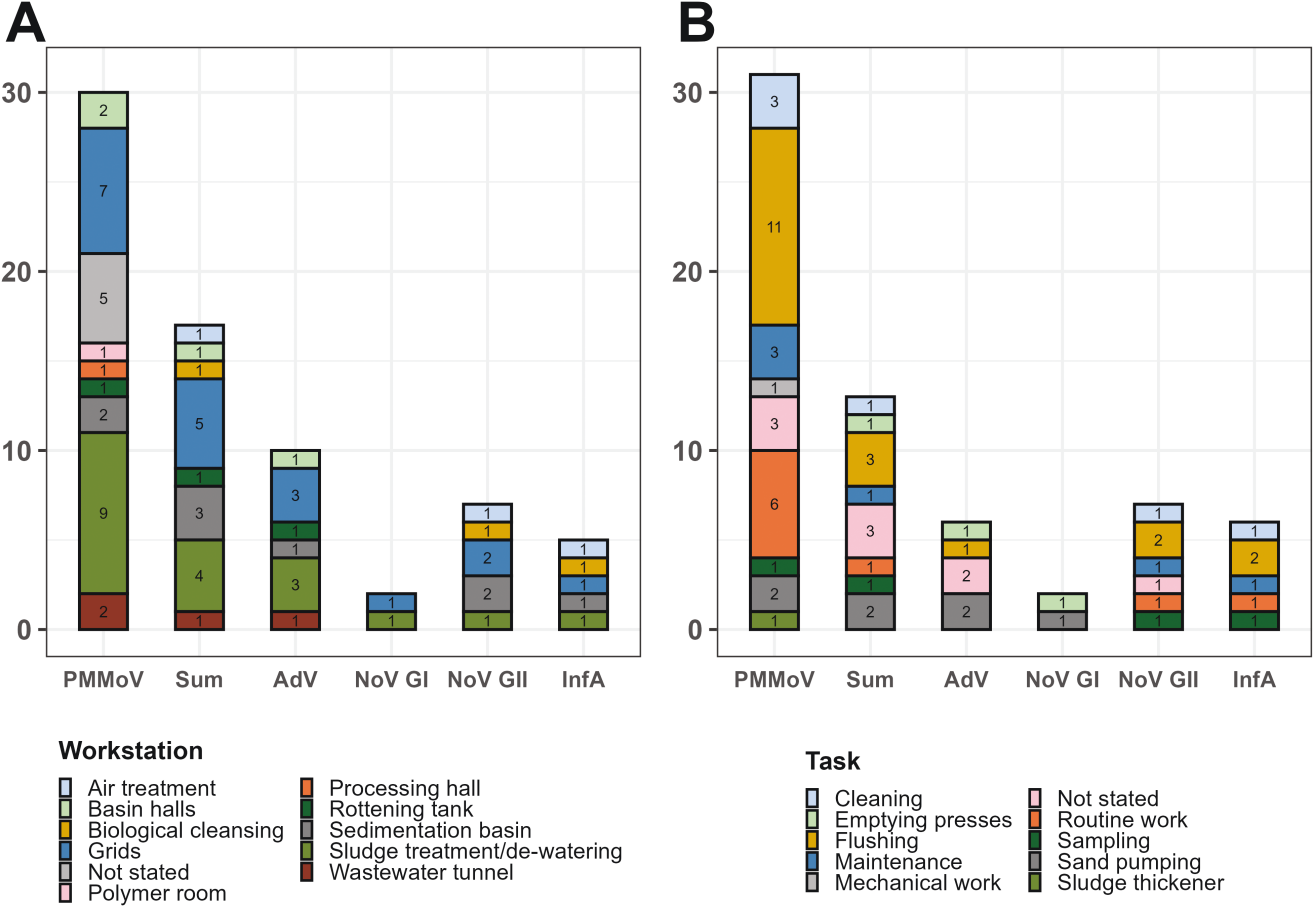
The workstations and tasks in wastewater treatment plants with detection of Pepper mild mottle virus (PMMoV) and Adenovirus (AdV), Norovirus GI and GII, Influenza A (InfA) and the sum of all viral pathogens (Sum) in personal air samples across all plants and seasons. (A) The absolute number of workstations grouped by positive samples and (B) the absolute number of workers’ tasks grouped by positive samples.

PMMoV was detected across all workstations, with highest detection rates observed at the grids, biological cleansing, sedimentation basins, and the sludge treatment/de-watering stations in both stationary (Figure 1A) and personal samples (Figure 2A), although no significant difference between workstations could be observed. Results from both PMMoV and pathogens in 14 samples revealed co-occurrence in 8 (57%) overall. In stationary samples, co-occurrence was observed for all pathogen-positive Coriolis samples (*n* = 3) and half of the CIS cassettes (2 out of 4). Among the 7 personal samples, approximately 43% (*n* = 3) showed co-occurrence between PMMoV and pathogens.

Workers with personal air samples positive for PMMoV, Adenovirus, Norovirus GI and GII and Influenza A, most frequently reported having worked at the grids, sludge treatment/de-watering, and sedimentation basin (Figure 2A). The most frequently performed task with positive samples was flushing (at the grids, sludge treatment/de-watering, basin halls) followed by routine work and maintenance, and sand pumping (performed at the grids station) (Figure 2B).

## Discussion

In this study, we detected human-pathogenic viruses transmissible via the fecal-oral and airborne routes; Adenovirus (AdV), Influenza A (InfA), Norovirus (NoV) GI, and GII, in air samples from Norwegian WWTPs during the winter and summer of 2022/2023. Among 82 samples, 18 tested positive for at least one of the targeted pathogens. No significant differences in concentration or detection rates were observed between plants, seasons, or between personal and stationary samples. Notably, all 4 pathogens were identified in winter, whereas only AdV and NoV GII were detected in summer. PMMoV was present across all plants, seasons, and workstations, in 67% of the samples, highlighting the aerosolization of viruses from sewage and the potential of PMMoV as a general indicator of viruses in WWTP air originating from wastewater.

### Aerosolized viruses in relation to other studies

The highest concentration observed for the most frequently detected pathogen, AdV at 460 gc/m^3^, was as much as 207-fold lower than the 95 179 gc/m^3^ reported by Masclaux et al. (2014) from Swiss WWTPs, and 5- and 15-fold lower than previous reports from Italian and Polish WWTPs (Carducci et al. 2016; Stobnicka-Kupiec et al. 2022). The 10% detection rate of NoV GII was noteworthy higher than in Danish, French, and Canadian WWTPs, where NoV GII was not detected, and in Switzerland, where only 2% of air samples were positive (Uhrbrand et al. 2011; Masclaux et al. 2014; Courault et al. 2017; Brisebois et al. 2018). A higher detection rate of 23% was found for NoV GII in Polish WWTP air (Stobnicka-Kupiec et al. 2022). Furthermore, we successfully detected InfA in 3 samples, which contrasts with findings from previous studies (Brisebois et al. 2018; Stobnicka-Kupiec et al. 2022). Our data revealed that PMMoV was present in the air across all plants, seasons, and workstations. PMMoV was monitored by Amato et al. (2023) in wastewater from the same plants and period as our study, and it is still undetected in Norwegian agriculture (personal communication, Dr. Zhibo Hamborg). Despite its distinctive morphology compared to most human-pathogenic viruses, PMMoV, as part of a larger bioaerosol particle, is likely to have an aerosolization potential similar to other viruses (Verreault et al. 2008). Repeated one-year measurements in wastewater have shown correlations between PMMoV concentrations, and those of human Adenovirus and Norovirus GII (Tandukar et al. 2020). Therefore, it is plausible that its presence can serve as a marker of aerosolized human pathogenic viruses originating from wastewater.

### Seasonal differences and gaps in detection of PMMoV and pathogens

Most pathogens were detected in winter when the detection rate of PMMoV in stationary CIS cassettes was lower than in summer. Human-pathogenic viruses, both respiratory and gastrointestinal, typically exhibit heightened prevalence during winter, increasing the virus concentration in wastewater (Grondahl-Rosado et al. 2014; Myrmel et al. 2015; Hyllestad et al. 2022), affecting the airborne levels. On the contrary, the presence of PMMoV in Norwegian wastewater depends on the population’s relatively stable dietary consumption, although seasonal variation due to differences in import cannot be excluded. Precipitation levels, snowmelt, and temperature affect wastewater composition and volume (Parkins et al. 2024). Higher winter precipitation and an abnormally dry June in 2023 (Norwegian Meteorological Institute and the Norwegian Broadcasting Corporation 2023) may explain the higher PMMoV detection in summer in stationary CIS cassettes, due to a more concentrated sewage (Goitom et al. 2024). An exposure assessment with only stationary CIS-cassettes would likely underestimate winter exposure risk, as all pathogens were detected in winter, but only AdV and NoV GII in summer. The air sampling methodology, placement of the sampler relative to the bioaerosol source, sampling time, and airflow also affect virus detection (Prussin et al. 2014; Pan et al. 2019). Of the total dataset, 33% of samples were negative for PMMoV. However, the detection frequency was equal in both personal and stationary CIS cassettes (67-68%), and highest in Coriolis (88%). All the plants had implemented strategies for exposure reduction such as sealing of the grids station and the sludge centrifuges, which were opened only during manual work. Stationary CIS cassettes were static, placed 1 –to 3 meters from the source, during periods of both high and low activity. The personal samplers were dynamic and varied in proximity to the wastewater and sludge, depending on the workers’ tasks. Coriolis, on the other hand, collected high-volume air samples during active tasks at open workstations, representing short periods with high bioaerosol generation. Low biomass of aerosolized viruses is a well-known challenge in air sampling (Prussin et al. 2014). CIS cassettes are designed to collect the inhalable fraction, making them more representative of workers’ potential exposure compared to high-volume samples. This may explain lower PMMoV and pathogen co-occurrence in CIS cassettes than Coriolis. As an ideal indicator, PMMoV should co-occur with pathogens, which was true for all pathogen-positive Coriolis samples (*n* = 3), but only 45% of the pathogen-positive CIS cassettes. Most PMMoV-negative and pathogen-positive samples were personal, and we cannot exclude the possibility that the pathogens originated from the workers. Although PMMoV and pathogens correlate in wastewater over time (Tandukar et al. 2020), concentrations can vary significantly within one day (Ahmed et al. 2021). Thus, timing is important to assess both exposure and co-occurrence. The lack of co-occurrence of PMMoV alongside pathogen-positive CIS-cassettes does not in itself exclude PMMoV as an indicator of aerosolized pathogenic viruses. It may rather reflect that the detection of extremely low levels of viruses is largely dependent on the sampled air volume, of which Coriolis exceeds CIS-cassettes. Negative samples may contain untargeted viruses (Brisebois et al. 2018) and recently, several indicators of fecal pollution in water have been suggested, such as CrAssphage (Park et al. 2020; Tandukar et al. 2020; Ahmed et al. 2021) and Tobacco Mosaic Virus (Tandukar et al. 2020). Comparison and validation of indicators and their correlation with pathogenic viruses in wastewater and bioaerosols are needed to simplify occupational exposure assessment in WWTPs. Alternative indicators can be identified from reviews of previous studies on wastewater-based epidemiology (Parra-Arroyo et al. 2023).

### ddPCR limit of detection relative to qPCR in other studies

Pathogenic viruses in our study ranged from 18 to 756 gc/m³ air, below the 1000 gc/m³ detection limit reported in qPCR studies (Brisebois et al. 2018; Stobnicka-Kupiec et al. 2022). With our LOD of 292 genome copies per sample (concentrated liquid or filter), we estimated virus concentrations down to 18 gc/m^3^ air. To the best of our knowledge, this study is the first to use ddPCR in WWTP air samples. Our results confirm the superior sensitivity of ddPCR over qPCR (Ciesielski et al. 2021) and suggest this method enables the detection of previously undetected pathogens, like InfA and NoV GII. ddPCR allows detection of viral targets in low volumes and airflow, more relevant of workers’ exposure assessment than high-volume sampling. Its application in other occupational settings may improve exposure assessment of low-abundant targets.

### Evaluation of the infection risk

The time-weighted average genome concentrations of pathogens in the personal samples peaked at 120 (AdV), 452 (InfA), 144 (NoV GI), and 226 gc/m^3^ (NoV GII). We employed a universal primer for Adenovirus, which excludes the distinction between respiratory and gastroenteric serotypes (Jothikumar et al. 2005), a common approach in bioaerosol studies (Masclaux et al. 2014; Stobnicka-Kupiec et al. 2022). Assessing infection risk from PCR data poses a dual challenge in occupational risk assessment: on one hand, specific primers are required to detect each unique virus, meaning samples might contain untargeted viruses and, therefore, the risk is underestimated. On the other hand, the quantified viral genome copies may originate from non-infectious, virions, potentially overestimating workers’ risk of infection. Furthermore, the minimum infective dose (MID) varies between viruses and serotypes, ranging from as little as 6 to 7 (Adenovirus serotype E) and 18 (Norovirus) viral particles to more than 1000 (SARS-Cov-2) (Yezli and Otter 2011; McCormick and Mermel 2021). Enveloped viruses tend to lose infectivity in wastewater more rapidly than non-enveloped ones, thus the proportion of infectious Influenza A in our study is uncertain (Gundy et al. 2008; Corpuz et al. 2020). Stobnicka-Kupiec and colleagues found that approximately 20% of the detected genome copies of NoV GII and AdV in WWTP air originated from intact capsids (Stobnicka-Kupiec et al. 2022). Assessing infectivity and capsid integrity is beyond the scope of this study. However, assuming 20% of the genome copies represent infectious viruses, the levels of AdV and NoV in the present study may be sufficient to cause disease in workers. High humidity in all plants, regardless of workstation and season, may promote bioaerosol droplets (≥5 µm) that can remain airborne for about 30 minutes and deposit in the trachea or nasal cavity (Poh et al. 2018; Joseph et al. 2022). If these particles carry gastrointestinal viruses, there’s a risk of ingestion and infection. Respiratory viruses like InfA may be less virulent in this size range, as optimal virulence occurs in drier air (Marr et al. 2019). However, viruses such as InfA and AdV can infect both the upper and lower airways, indicating possible pathogenicity in all particle sizes (Thomas 2013). The presence of PMMoV in various-sized bioaerosols could thus indicate potentially infectious viruses, irrespective of particle size. Given the uncertainty around preserved infectivity and required infectious dose, we propose that all pathogen and PMMoV positive samples should be considered potential risks, regardless of concentration.

### Identification of high-risk workstations and tasks

PMMoV and pathogens were most frequently detected in both stationary and personal samples associated with the grids, sludge and de-watering stations, and sedimentation basins/basin halls. These workstations are equivalent to those with the highest detection rates and concentrations reported by Stobnicka-Kupiec et al. (2022). Moreover, these workstations have previously been associated with an increased risk of symptoms such as soft stools, throat irritation, unusual tiredness, cough with phlegm, and diarrhea (Thorn et al. 2002). Further, they are associated with the tasks most frequently performed by workers with positive samples, i.e. flushing, maintenance, cleaning, and sand pumping. All these tasks involve direct contact with sludge or the potential for aerosolization of sewage.

### Limitations and recommendations for future studies

This study has certain limitations. The number of participating workers varied across plants (n = 10/20/1 in plant A/B/C, respectively) and between seasons (*n* = 21/6 in winter/summer, respectively). This variability in sample size limited our ability to assess differences between plants and seasons based on personal samples. However, by collecting information on the workers’ daily activities, we could identify high-risk workstations and tasks that may be relevant and transferable to other WWTPs. Our assessment of exposure to human pathogenic viruses and their correlation with PMMoV as an indicator is solely based on the detection of AdV, InfA, NoV GI, and NoV GII. We acquired results on both PMMoV and the selected pathogens in 14 samples (Figure 1B). This sample number is too small for robust conclusions on PMMoV’s applicability as an indicator of aerosolized viruses with human waste origin. Furthermore, fecal indicators such as PMMoV are associated with human feces and cannot be expected to correlate with human-pathogenic viruses shed through vomit, respiratory phlegm, urine, genitals, or blood. We recommend future studies to include a larger variety of pathogens of both gastrointestinal, respiratory, and genital origin. This can be accomplished by developing multiplexed assays for ddPCR/qPCR, Luminex, etc. By using RT-ddPCR, the LOD per m^3^ can be lowered further, due to fewer dilution steps. Lastly, PMMoV is a plant virus, and in regions with crop infections, its levels in wastewater may result from agricultural runoff in addition to feces. Future studies using this virus as an indicator should carefully assess the impact of such confounding sources, for improved interpretation of occupational risk.

## Conclusion

Our results demonstrate that workers in Norwegian WWTPs are exposed to airborne pathogenic viruses, although exposure levels are generally low. Despite aerosol reduction measures, there is a risk of higher exposure at specific workstations, such as the grids, biological cleansing pools, sedimentation basins, and sludge treatment stations, particularly during tasks such as flushing, cleaning, and maintenance. This highlights the need for proper personal protective equipment (face masks and eye protection) and relevant vaccines to safeguard WWTP workers’ health. Our results further demonstrate that using a variety of samplers, including low-volume CIS cassettes and high-volume Coriolis, combined with sensitive detection methods such as ddPCR, provides a comprehensive assessment of exposure. PMMoV shows potential in occupational risk assessment as a general indicator of viruses with human waste origin in WWTP air. However, further validation of PMMoV and other indicators is recommended to validate the correlation with pathogens in both wastewater and the aerosol. Finally, a detailed characterization of the airborne virome and associated disease symptoms related to operational tasks and workstations would be valuable for assessing the overall impact of viral particles on workers’ health to better target health and safety strategies.

## Supplementary material

Supplementary material is available at *Annals of Work Exposures and Health* online.

wxaf020_suppl_Supplementary_Tables_S1-S5_Figures_S1

## Data Availability

The data underlying this article will be shared at reasonable request to the corresponding author.

## Statements

SIKT KI chat (supports ChatGPT 3.5 and 4) were used to troubleshoot R and STATA scripts and for inspiration in rephrasing the manuscript. However, all analyses and texts are written and produced by the authors.
