# Supplementary material for: Pepper mild mottle virus as a potential indicator of occupational exposure to airborne viruses in wastewater treatment plants
Source: Ann Work Expo Health. 2025 May 24;69(5):495–509. doi: 10.1093/annweh/wxaf020 (PMC12208365; doi:10.1093/annweh/wxaf020)
Supplement: wxaf020_suppl_Supplementary_Tables_S1-S5_Figures_S1 [file wxaf020_suppl_supplementary_tables_s1-s5_figures_s1.pdf]

Pepper mild mottle virus as a potential indicator of occupational exposure to airborne viruses in wastewater treatment plants – Supplementary information

Anna Jacobsen Lauvås[1,3], Pål Graff[4], Anani K. Afanou[1], Caroline Duchaine[2], Marc Veillette[5], Mette Myrmel[3], Anne Straumfors[1*]

[1]Department of Occupational Toxicology, National Institute of Occupational Health, Gydas vei 8, 0363 Oslo, Norway

[2]Département de Biochimie, de Microbiologie et de Bio-informatique, Faculté des Sciences et de Génie, Université Laval, Pavillon Alexandre-Vachon, 1045 Av. de la Médecine, Québec, QC G1V 0A6, Canada Québec, Canada

[3]Virology unit, Department of Paraclinical Sciences, Faculty of Veterinary Medicine, Norwegian University of Life Sciences, Elizabeth Stephansons v. 15, 1433 Ås, Norway

[4]Department of Chemical Work Environment, National Institute of Occupational Health, Gydas vei 8, 0363 Oslo, Norway

[5]Centre de Recherche, Institut Universitaire de Cardiologie et de Pneumologie-Université Laval, 2725 Ch Ste-Foy, Québec, QC G1V 4G5, Canada

*Corresponding author: anne.straumfors@stami.no

Table S1: Primers and probe sequences for the detection of viruses

Virus	Primers and probes	Sequences	Reference
**Human AdV A-F**	JTXVF	5’ GGACGCCTCGGAGTACCTGAG 3’	Jothikumar et al. (2005)
	JTXVR	5’ ACIGTGGGGTTTCTGAACTTGTT 3’	
	JTXVP	6-FAM 5’ CTGGTGCAGTTCGCCCGTGCCA 3’- MGBNFQ	
**InfA**	InfA F	5’ GACCRATCCTGTCACCTCTGAC 3’	Brisebois et al. (2018)
	InfA R	5’ AGGGCATTYTGGACAAAKCGTCTA 3’	
	InfA P	5’ TGCAGTCCTCGCTCACTGGGCACG 3’-MGBNFQ	
**NoV GI**	Noro GI F	5’ CGYTGGATGCGNTTYCATGA 3’	Brisebois et al. (2018)
	Noro GI R	5’ CTTAGACGCCATCATCATTYAC 3’	
	Noro GI P	6-FAM 5’ AGATYGCGATCYCCTGTCCA 3’ -MGBNFQ	
**NoV GII**	Noro GII F	5’ CARGARBCNATGTTYAGRTGGATGAG 3’	Brisebois et al. (2018)
	Noro GII R	5’ TCGACGCCATCTTCATTCACA 3’	
	Noro GII P	6-FAM 5’ TGGGAGGGCGATCGCAATCT 3’ -MGBNFQ	
**PMMoV**	PMMV-FP1	5’ GAGTGGTTTGACCTTAACGTTTGA 3’	Zhang et al. (2006)
	PMMV-RP1	5’ TTGTCGGTTGCAATGCAAGT 3’	
	PMMV-Probe1	6-FAM 5’ CCTACCGAA/ Zen /GCAAATG 3’ - 3IABkFQ	

Table S2: The minimum estimated air concentration of viruses in air volumes up to 8 m^3 based on an assay limit of detection of 1 positive droplet. The estimated concentrations in genome copies per m^3 air are given for both one and the average of two technical replicates.

**Air (m^3)**	**Gc/m^3**	
	1 rep.	2 reps.
**1**	292	146
**2**	146	73
**4**	73	36.5
**8**	36.5	18

Table S3: Statistical summary of sampling time and air volume in the CIS, Coriolis, and Impinger samplers. The air volume and sampling time are presented as the arithmetic mean and range for all samples collected (N)

		CIS Personal	CIS Stationary	Coriolis
	N	31	30	26
Air volume (m^3)	Min - max<br>Mean ± SD	1 – 1.5<br>1.3 ± 0.1	0.9 – 8.1<br>1.7 ± 1.4	2 - 8<br>5.1 ± 1.9
Sampling time (h:min)	Min - Max<br>Mean ± SD	4:35 - 7:20<br>6:07 ± 0:07	3:46 - 7:58<br>6:08 ± 1:13	0:10 - 0:40<br>0:25 ± 0:10

Table S4: Liquid recovery of Coriolis samples, relative humidity (%RH) and indoor and outdoor temperature at the time of sampling.

**Plant and season**	**Liquid recovery (%)**		**%RH**		**Temperature (°C)**	
	Mean (Min. – Max.)	n	Mean (Min. – Max.)	n	Indoor (Mean ± SD)	Outdoors
**A**						
Summer	92 (87 – 99)	5	68 (52 – 77)	8	19 ± 1	20
Winter	121	1	57 (25 – 85)	3	16 ± 3	-2
**B**						
Summer	74 (63 – 81)	3	44 (37 – 55)	4	24 ± 4	23
Winter	72 (67 – 76)	2	32 (27 – 35)	4	20 ± 2	-2
**C**						
Winter	-	-	21 (20 – 23)	4	18 ± 1	-3
Total	86 (63 – 121)	18	49 (20 – 85)	23	19 ± 3	

*Table S5: Stationary CIS cassettes and Coriolis samples positive for Adenovirus, Influenza A, Norovirus GI and GII and PMMoV across plants and seasons. The data are presented as detection rates (positive count and % of total tested samples, N). ND = Not detected. * Significant difference between seasons ($p < 0.05$)*

	**Pos/N (%)**						
	**Target**	**All samples**	**Summer**	**Winter**	**Plant A**	**Plant B**	**Plant C**
Stationary CIS	AdV	1/29 (3)	1/15 (7)	0/14 (ND)	0/14 (ND)	1/10 (10)	0/5 (ND)
	InfA	1/26 (4)	0/12 (ND)	1/14 (7)	0/11 (ND)	0/10 (ND)	1/5 (20)
	NoV GI	0/27 (ND)	0/13 (ND)	0/14 (ND)	0/12 (ND)	0/10 (ND)	0/5 (ND)
	NoV GII	3/27 (11)	1/13 (8)	2/14 (14)	2/12 (17)	1/10 (10)	0/5 (ND)
	Sum pathogens	5/29 (17)	2/15 (13)	3/14 (21)	2/14 (14)	2/10 (20)	1/5 (20)
	PMMoV	19/28 (68)	13/15 (87) *	6/13 (46) *	11/14 (79)	5/9 (56)	3/5 (60)
Coriolis	AdV	5/20 (25)	0/2 (ND)	5/18 (28)	2/9 (22)	2/7 (29)	1/4 (25)
	InfA	0/5 (ND)	-	0/5 (ND)	0/1 (ND)	0/3 (ND)	0/1 (ND)
	NoV GI	0/4 (ND)	-	0/4 (ND)	-	0/3 (ND)	0/1 (ND)
	NoV GII	1/8 (12)	0/2 (ND)	1/6 (17)	0/4 (ND)	0/3 (ND)	1/1 (100)
	Sum pathogens	6/20 (30)	0/2 (ND)	6/18 (33)	2/9 (22)	2/7 (29)	2/4 (50)
	PMMoV	14/16 (88)	6/7 (86)	8/9 (89)	6/7 (86)	6/7 (86)	2/2 (100)

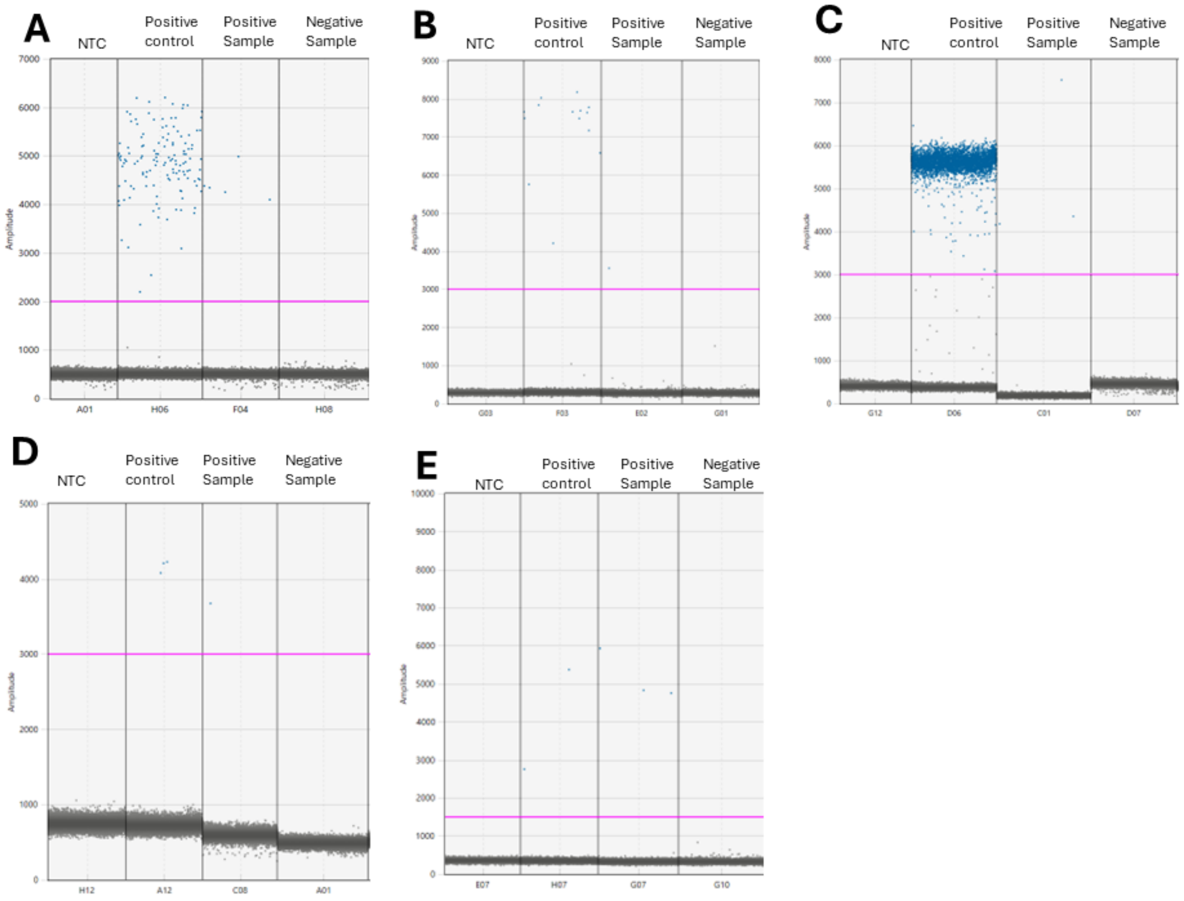

Figure S1: Representative ddPCR amplitude plots for No template control (NTC), positive control, an unknown positive sample, and an unknown negative sample for PMMoV (A), AdV (B), InfA (C), Nov GI (D), and NoV GII (E).

References

Brisebois E, Veillette M, Dion-Dupont V, Lavoie J, Corbeil J, Culley A, Duchaine C. 2018. Human viral pathogens are pervasive in wastewater treatment center aerosols. J Environ Sci (China). 67:45-53.

Jothikumar N, Cromeans TL, Hill VR, Lu X, Sobsey MD, Erdman DD. 2005. Quantitative real-time pcr assays for detection of human adenoviruses and identification of serotypes 40 and 41. Appl Environ Microbiol. 71(6):3131-3136.

Zhang T, Breitbart M, Lee WH, Run JQ, Wei CL, Soh SW, Hibberd ML, Liu ET, Rohwer F, Ruan Y. 2006. Rna viral community in human feces: Prevalence of plant pathogenic viruses. PLoS Biol. 4(1):e3.
